# Quality assessment of Brazilian olive oils by GC×GC–MS and chemometrics

**DOI:** 10.1007/s00216-026-06321-8

**Published:** 2026-01-27

**Authors:** Andre Cunha Paiva, Glaucimar Alex Passos de Resende, Luidy Darllan Barbosa, Daniel Lucas Dantas Freitas, Guilherme Post Sabin, Leandro Wang Hantao

**Affiliations:** 1 Instituto de Química (IQ), Universidade Estadual de Campinas, Rua Monteiro Lobato, 270, Campinas, SP 13083-862 Brazil; 2 Instituto Nacional de Ciência e Tecnologia (INCTBio), Campinas, SP Brazil; 3 OpenScience, Campinas, SP Brazil; 4 Núcleo Interdisciplinar de Planejamento Energético (NIPE), Campinas, SP Brazil

**Keywords:** PLS-DA, Extra virgin olive oil, Comprehensive two-dimensional gas chromatography, Mass spectrometry, Food authenticity, Adulteration

## Abstract

Virgin olive oil quality assessment is critical for consumer protection and industry regulation. Fraudulent activities involving virgin olive oil are common worldwide. In Brazil, recent nationwide inspections have revealed recurrent cases of fraud, including the sale of adulterated or mislabeled olive oils. As a result, the Brazilian federal agency has suspended the commercialization of numerous brands and seized large quantities of products that failed to meet the official quality requirements. This study presents an automated analytical workflow for classifying Brazilian virgin olive oils based on their volatile organic compound (VOC) profiles. Headspace solid-phase microextraction (HS-SPME) and comprehensive two-dimensional gas chromatography-mass spectrometry (GC×GC–MS) were used to analyze 215 certified virgin olive oil samples, categorized as either defective (VOO, virgin and LVOO, lampante virgin) or non-defective (EVOO, extra virgin), and 21 undisclosed oils (UNKN). Chemometric modeling using partial least squares-discriminant analysis (PLS-DA) was applied to the resulting data, which comprised 108 recurring VOCs. The PLS-DA model successfully differentiated between the two quality classes (defective and non-defective) with a predictive accuracy of 91% based on the external validation set. Key chemical markers driving the classification were identified. Non-defective oils were characterized by higher levels of C_5_ and C_6_ aldehydes and alcohols from the lipoxygenase (LOX) pathway, such as (E)-2-hexenal, associated with positive green and fruity notes. Conversely, defective oils showed higher levels of compounds such as nonanal and acetic acid, linked to rancidity and other off-flavors. Finally, the model was used to predict the quality of 21 previously undisclosed samples. This instrumental approach demonstrates a powerful and reliable alternative for forensic analysis of olive oils, generating models that can be interpreted in sensory terms.

**Graphical Abstract:**

**Figure:**
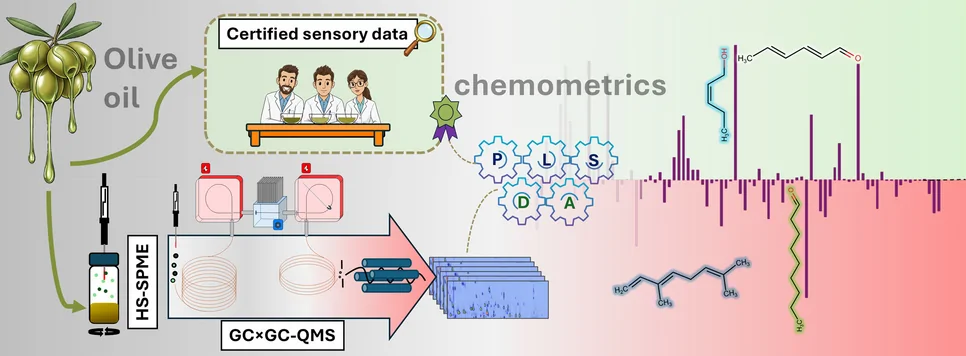

**Supplementary Information:**

The online version contains supplementary material available at https://doi.org/10.1007/s00216-026-06321-8.

## Introduction

Olive oil is a highly appreciated food commodity, widely recognized for its sensory characteristics, versatility, and significant health benefits [1, 2]. As a foundation of the Mediterranean diet, its cultural and geopolitical importance was formally acknowledged by its inclusion in UNESCO’s list of Intangible Cultural Heritage [3]. Unlike common vegetable oils derived from seeds, the final product is obtained directly from the fruit of the olive tree (*Olea europaea*), giving it a distinct chemical composition [4].

The prestige and price of premium olive oil, particularly extra virgin olive oil (EVOO), make it a primary target for economically motivated food fraud. The most common fraudulent practice in EVOO is adulteration. This practice represents 22% of food fraud instances and includes food product modification by removing, adding, substituting, or covering substances to mislead consumers [5]. This practice affects not only the commodity’s value but also food safety and quality and undermines the integrity of the market. Olive oil adulteration often involves mixing EVOO with cheaper oils or mislabeling its geographical origin. Virgin olive oil can be classified into EVOO, virgin (VOO), or lampante virgin olive oil (LVOO) [6]. Another possible food fraud scheme involves substituting or mixing EVOO with lower-grade VOO and LVOO.

The authenticity of olive oil is a challenge for the market, and analytical tools offer a great strategy to combat this fraud. The quality of olive oil requires a trained sensory panel to grade it [2, 7]. Alternatively, analytical techniques can be used to authenticate olive oil quality grades [8, 9], geographical location [10, 11], and cultivars [4, 12]. Spectroscopic methods like Fourier-transform medium infrared (FT-IR) [7], near-infrared (NIR) [13], and fluorescence spectrometry [14] are often used for rapid screening. Furthermore, fatty acid profiles can reveal olive oil fraud [15]. However, despite the advantages of these techniques, they cannot fully assess chemical composition or identification, which may limit the interpretability of the chemometric model.

Therefore, comprehensive two-dimensional gas chromatography coupled with mass spectrometry (GC×GC–MS) can offer a detailed chemical analysis capable of detecting subtle adulteration [16–18]. Among the variety of advantages, this technique offers higher peak capacity and increased sensitivity, allowing for more confident chemical identification [16]. This is particularly relevant because the volatile organic compound (VOC) profile of virgin olive oil is a key indicator of both authenticity and quality. In extra virgin olive oil (EVOO), the volatile fraction constitutes a distinctive “aroma blueprint,” a specific pattern of odor-active compounds that defines its sensory and chemical identity [19].

GC×GC–MS produces large and information-rich datasets, requiring chemometric methods for efficient interpretation of the chemical data. Chemometrics provides multivariate methods for extracting meaningful information and building classification models. Chemometric methodologies applied to olive oil data can be found in the literature, with example methods such as linear discriminant analysis (LDA) [4, 20], support vector machines (SVM) [20], data-driven soft independent modeling of class analogy (DD-SIMCA) [13], parallel factor analysis (PARAFAC) [13, 21], and partial least squares discriminant analysis (PLS-DA) [4, 21, 22].

In Brazil, this issue has attracted growing attention from the Ministry of Agriculture, Livestock and Food Supply (MAPA), the federal agency responsible for regulating food quality and authenticity. In 2025, recent nationwide inspections have revealed recurrent cases of fraud, including the sale of adulterated or mislabeled olive oils. As a result, MAPA has suspended the commercialization of numerous brands and seized large quantities of products that failed to meet the official quality and identity requirements. MAPA has strengthened analytical monitoring through the Federal Laboratories for Agricultural Defense (LFDA), which perform chemical and physical analyses, including sensory and chromatographic analyses. Consequently, advanced and automated chromatographic methods are required to support regulatory surveillance and consumer protection by providing reliable methods for authentication, traceability, and detection of adulteration in olive oils, while generating models that can be interpreted in sensory terms.

In this context, the present study aims to assess virgin olive oil quality using a classification model trained on the volatile organic compound (VOC) profiles of defective (VOO and LVOO) and non-defective (EVOO) samples. Headspace solid-phase microextraction (HS-SPME) was employed to extract VOCs from 236 olive oil samples, and the volatile fraction was analyzed by GC×GC–MS equipped with a thermally independent solid-state modulator. A total of 215 certified virgin olive oil samples from MAPA were used to calibrate and validate the PLS-DA model, achieving a predictive accuracy of 91% on an external validation set. Finally, the model was applied to classify 21 undisclosed olive oil samples. This study represents a significant achievement, as it encompasses a large and diverse set of real-world samples and demonstrates the practical potential of GC×GC–MS combined with chemometrics for forensic applications in olive oil authentication.

## Material and methods

### Samples

A total of 215 certified virgin olive oil samples were provided by the Ministry of Agriculture, Livestock, and Food Supply (MAPA – Brazil). A total of 137 samples were classified as extra virgin (EVOO), 47 as virgin (VOO), and 31 as lampante virgin (LVOO) olive oils, according to the criteria of Normative Instruction No. 1 of 2012 from MAPA (see Electronic Supplementary Material Table S1) [6]. An additional 21 samples consisted of undisclosed olive oils, hereafter referred to as “unknown samples” (UNKN). Once received, all samples were stored in a light-protected environment at controlled temperatures for 6 months before analysis.

The samples in this study were olive oils produced in the year 2024, and the conclusions of this study are restricted to the samples evaluated from this period. Furthermore, interesting studies have already demonstrated that the year of production impacts predictive models for olive oil samples [23].

### Solid-phase microextraction

VOCs were extracted using HS-SPME. A 2-cm-long SPME fiber coated with 50/30 µm divinylbenzene/carboxen/polydimethylsiloxane (DVB/CAR/PDMS) (Merck Millipore – USA) was used to sample the analytes. Twenty-milliliter glass vials and magnetic stainless steel screw caps with PTFE/Silicone septa were also used for the extractions.

The SPME fiber was conditioned according to the manufacturer’s instructions before use. The HS-SPME extraction step was performed using a TriPlus RSH autosampler (Thermo Fisher Scientific – USA). The DVB/CAR/PDMS sorbent phase was chosen based on previous studies [4, 18, 24–26]. A 0.25 g aliquot of each olive oil sample was placed into a 20 mL headspace vial. The extraction was performed at 250 rpm agitation. The vials were pre-equilibrated at 40 °C for 10 min. Subsequently, the SPME extraction was conducted at 40 °C for 50 min. The analytes were thermally desorbed from the SPME coating for 1 min.

### GC×GC-QMS

The experiments were conducted on a TRACE 1300 gas chromatograph coupled to an ISQ single-quadrupole mass spectrometer (QMS) (Thermo Fisher Scientific).

The GC was equipped with a split/splitless (SSL) injector held at 240 °C and operated in splitless mode for 1 min. The carrier gas was helium at a constant flow of 1.0 mL/min. The single GC oven was programmed from 40 to 220 °C at 3 °C/min, with a final isotherm of 5 min.

Thermal modulation was performed using a consumable-free solid-state modulator, SSM1800 (J&X Technologies – China). The “polar × non-polar” column setup consisted of a SUPELCOWAX 10 column (30 m × 0.25 mm × 0.25 µm) (Merck Millipore) connected to a HP-1 column (1 m × 0.1 mm × 0.1 µm) (Agilent Technologies – USA). Siltite micro-unions (Trajan Scientific and Medical – Australia) were used for connecting these two capillary columns with the SV modulation capillary. The modulation period was 6 s. The cold trap was kept at − 51 °C. The entry and exit zones were heated at 220 °C and 250 °C, respectively.

The transfer line and the ion source were maintained at 260 °C and 275 °C, respectively. Electron ionization at 70 eV was used in all experiments. The acquisition rate was 28 spectra/s with a mass range of 40–350 m*/z*. Chromeleon software v7.3.2 (ThermoFisher Scientific) was used for instrument control and data acquisition.

### Identification

The GC IMAGE software (GC Image, LLC – USA) was used for peak detection, template matching, and alignment.

Composite chromatograms were generated using INVESTIGATOR software (GC Image) to highlight the volatile organic compounds (VOCs) that contributed most to distinguishing the two classes of interest: defective and non-defective olive oils.

Feature annotation was performed using a minimum mass spectral similarity match of 80% and a retention index tolerance of ± 20 units. The linear temperature-programmed retention index (LTPRI) was calculated according to the equation described by van den Dool and Kratz [27]. Experimental entries were compared with the NIST20 mass spectral library (National Institute of Standards and Technology, USA) using standard polar column reference values for LTPRI filtering.

Odor descriptors for the identified compounds were obtained from the literature [1, 16, 18, 24, 28–30].

### Data processing

The peak tables were exported from GC Image in “.csv” format and concatenated to form three data matrices *X*, containing all independent variables for modeling. The first dataset comprised 142 virgin olive oil samples and 108 features (*X*_cal_, 142 × 108), and it was used for PLS-DA training and internal cross-validation. The second data matrix (*X*_val_, 73 × 108) was used for external validation. The duplex algorithm was used for data splitting to avoid overfitting [31, 32]. The third data consisted of the 21 undisclosed samples (*X*_unkn_, 21 × 108).

Chemometric analysis was performed in MATLAB R2021b (MathWorks, USA). The data was pre-processed using autoscaling in PLS Toolbox 9.0 (Eigenvector Research – USA). External validation using an independent data set (*X*_val_) is crucial for assessing the model’s generalization ability and ensuring it accurately predicts new samples not part of the original dataset, thereby providing a more reliable indication of its true predictive power and preventing overfitting to the training dataset.

Data processing using PLS-DA enabled correlating the peak areas of VOCs in the measurements with the two classes of virgin olive oils studied (the property of interest). In this study, values of 0 (zero) were assigned to measurements from extra virgin olive oil (EVOO) samples (non-defective oils), and values of 1 (one) were assigned to measurements from virgin olive oil (VOO) and lampante virgin olive oil (LVOO) (defective oils).

The model for correlating experimental data (*X*) with the property of interest (*y*) was constructed, and the number of latent variables (LV) was selected based on analyses of mean calibration and cross-validation errors (Venetian blind method).

## Results and discussion

### HS-SPME-GC×GC-QMS

**Fig. 1:**
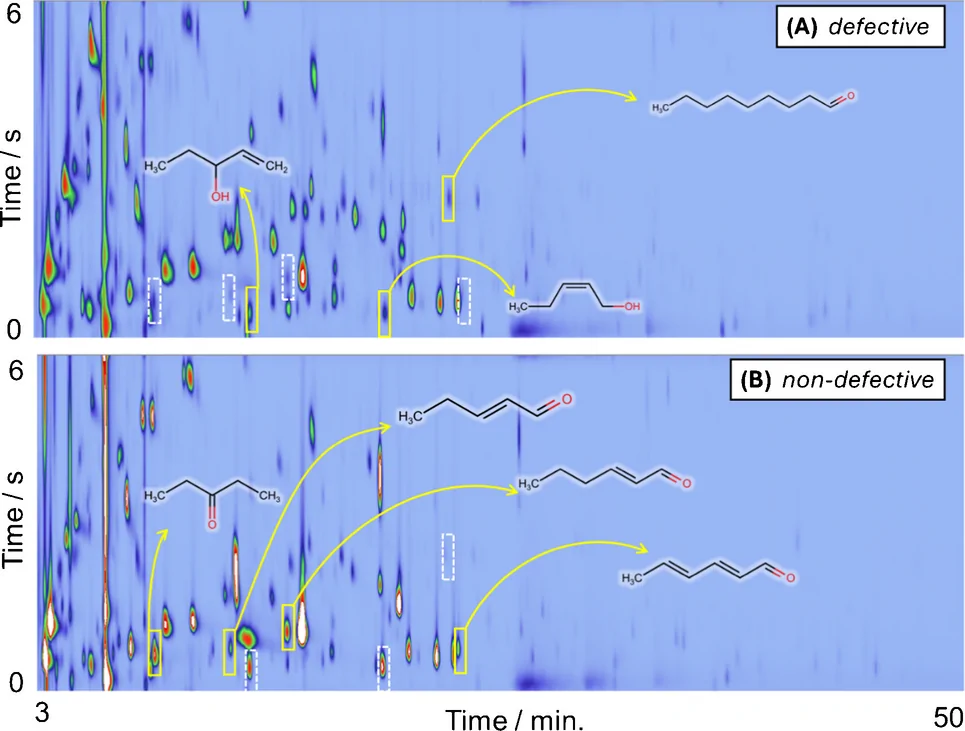
Composite-class images of **A** defective and **B** non-defective virgin olive oil groups. The chemical structures of some of the most informative analytes across the samples for the PLS-DA model are highlighted

Composite 2D chromatograms, a function available in INVESTIGATOR software, were generated from the MS detection channel by analyzing individual samples and combining them into images representing the volatile profiles of two classes: defective oils (virgin and lampante virgin) and non-defective oils (extra virgin). The resulting composite chromatograms (Fig. 1) for the two classes (Fig. 1A – defective, Fig. 1B – non-defective) reveal readily perceptible differences in their VOC profiles.

Analysis of 215 virgin olive oils yielded approximately 108 recurring VOCs, detailed in Table 1. The identified analytes belong to several chemical classes, including hydrocarbons (terpenes), esters, ketones, carboxylic acids, and alcohols—consistent with previous studies that evaluated olive oil volatiles [1, 8, 17, 18, 24, 25, 29, 30, 33]. To ensure that all chemical information obtained from the VOC profile of the virgin olive oil samples is adequately interpreted, a chemometric approach was employed to interpret the data and build a reliable classification model robustly.

**Table 1:** Analytes identified in the volatile and semi-volatile fraction of defective (VOO and LVOO) and non-defective (EVOO) virgin olive oil samples. ID levels stand for proposed levels for the reliability of compound identification, and more information about it is available at [34]

Analyte identity	M.F	ID level	CAS	Sensory attribute
(1-methylethyl)-benzene	C_9_H_12_	2	98–82-8	-
(1α,2β,4β)−1,2,4-trimethyl-cyclohexane	C_9_H_18_	3	7667–60-9	-
(2-phenylcyclopropyl)methanol	C_10_H_12_O	3	29667–46-7	-
(5E)−3-ethyl-1,5-octadiene ^Ψ^	C_10_H_18_	2	-	**-**
(E)- 6,10-dimethyl-5,9-undecadien-2-one	C_13_H_22_O	2	3796–70-1	-
(E)−2-heptenal ^Ψ^	C_7_H_12_O	3	18829–55-5	**Fatty, almond-like, green**
(E)−2-hexen-1-ol ^Ψ^	C_6_H_12_O	2	928–95-0	**Green grass and leaves**
(E)−2-hexenal ^Ψ^	C_6_H_10_O	2	6728–26-3	**Bitter almond, green**
(E)−2-penten-1-ol	C_5_H_10_O	3	1576–96-1	**Mushroom, earthy**
(E)−2-pentenal ^Ψ^	C_5_H_8_O	3	1576–87-0	**Pungent, apple-like**
(E)−3-hexen-1-ol	C_6_H_12_O	2	544–12-7	**Banana, fresh, green grass, leaf, cut grass, herbal**
(E)−3-hexenal ^Ψ^	C_6_H_10_O	2	4440–65-7	**Butter, green, leaf, grassy, vegetables, herbal**
(E)−4,8-dimethylnona-1,3,7-triene	C_11_H_18_	3	19945–61-0	**-**
(E)−7-pentadecene	C_15_H_30_	3	-	-
(E)-beta-ocimene ^Ψ^	C_10_H_16_	3	3779–61-1	-
(E)-decahydro-naphthalene ^Ψ^	C_10_H_18_	2	493–02–7	-
(E,E)−2,4-heptadienal ^Ψ^	C_7_H_10_O	2	4313–03–5	**Fatty, green, oily**
(E,E)−2,4-hexadienal ^Ψ^	C_6_H_8_O	2	142–83-6	**Fresh, green**
(E,E)-penta-2,4-dienal	C_5_H_6_O	3	764–40-9	-
(Z)−1,2-dimethyl-cyclohexane	C_8_H_16_	3	2207–01–4	-
(Z)−2-hexen-1-ol	C_6_H_12_O	2	928–94-9	**Green grass and leaves**
(Z)−2-hexenal	C_6_H_10_O	3	505–57-7	**Fruity, bitter almonds, sharp, astringent, apple-like, green, grass**
(Z)−2-penten-1-ol ^Ψ^	C_5_H_10_O	2	1576–95-0	**Green, plastic, rubber, almond**
(Z)−3-hexen-1-ol	C_6_H_12_O	2	928–96-1	**Banana, fresh, grass**
(Z)−3-hexen-1-ol acetate ^Ψ^	C_8_H_14_O_2_	3	3681–71-8	**Sweet**
(Z)−3-hexenal	C_6_H_10_O	3	6789–80-6	**Green, grassy**
1,2,3-trimethyl-benzene	C_9_H_12_	3	526–73-8	-
1,2,4,5-tetramethyl-benzene	C_10_H_14_	2	95–93-2	-
1,2,4-trimethyl-benzene ^Ψ^	C_9_H_12_	2	95–63-6	**-**
1,2,4-trimethyl-cyclohexane	C_9_H_18_	3	2234–75-5	-
1-butanol	C_4_H_10_O	2	71–36-3	**Winey**
1-ethyl-2,4-dimethyl-benzene	C_10_H_14_	3	874–41-9	-
1-ethyl-2-methyl-benzene	C_9_H_12_	2	611–14-3	-
1-ethyl-3,5-dimethyl-benzene	C_10_H_14_	2	934–74-7	-
1-ethyl-3-methyl-benzene	C_9_H_12_	2	620–14-4	-
1-hexanol ^Ψ^	C_6_H_14_O	3	111–27-3	**Fruity, banana, soft**
1-methoxy-hexane	C_7_H_16_O	3	4747–07-3	-
1-methyl-3-propyl-benzene ^Ψ^	C_10_H_14_	2	1074–43-7	-
1-nonylcycloheptane	C_16_H_32_	3	-	-
1-octanol ^Ψ^	C_8_H_18_O	2	111–87-5	**Moss, nut, mushroom**
1-pentanol	C_5_H_12_O	3	71–41-0	**Sweet, pungent, fusty, mold, vinegary**
1-penten-3-ol ^Ψ^	C_5_H_10_O	3	616–25-1	**Pungent, grass, green, olive fruit**
1-penten-3-one ^Ψ^	C_5_H_8_O	3	1629–58-9	**Mustard**
2,2,4-trimethyl-pentane ^Ψ^	C_8_H_18_	3	540–84-1	**-**
2,4-dimethyl-hexane	C_8_H_18_	3	589–43-5	-
2,6-dimethyl-heptane	C_9_H_20_	3	1072–05-5	-
2,6-dimethyloctane	C_10_H_22_	3	2051–30-1	-
2,6-dimethyl-pyrazine	C_6_H_8_N_2_	2	108–50-9	-
2,6-dimethyl-undecane ^Ψ^	C_13_H_28_	3	17301–23-4	-
2-butyl-1,1,3-trimethyl-cyclohexane	C_13_H_26_	3	54676–39-0	-
2-carene epoxide	C_10_H_16_O	3	20053–58-1	-
2-ethyl-1-hexanol ^Ψ^	C_8_H_18_O	2	104–76-7	-
2-ethyl-furan	C_6_H_8_O	2	3208–16-0	**-**
2-ethylhexyl acrylate	C_11_H_20_O_2_	2	103–11-7	-
2-ethylhexyl propanoate	C_11_H_22_O_2_	3	-	-
2-ethyl-oxetane	C_5_H_10_O	3	-	-
2-hydroxy-2-methyl-propanenitrile	C_4_H_7_NO	3	75–86-5	-
2-isopropyl-4-methyl-2,5-dihydrooxazole ^Ψ^	C_7_H_13_NO	3	111209–47-3	-
2-methyl-decane	C_11_H_24_	2	6975–98-0	-
2-methyl-heptane	C_8_H_18_	3	592–27-8	-
2-nitro-ethanol propionate	C_5_H_9_NO_4_	3	5390–28-3	-
2-propanone	C_3_H_6_O	3	67–64-1	-
3,4-dimethyl-heptane ^Ψ^	C_9_H_20_	3	922–28-1	-
3,5-dimethyl-octane	C_10_H_22_	3	15869–93-9	-
3,7,11-trimethyl-1-dodecanol	C_15_H_32_O	3	6750–34-1	-
3-methyl-1-butanol	C_5_H_12_O	2	123–51-3	**Malty, ethereal**
3-methyl-nonane	C_10_H_22_	2	5911–04–6	-
3-methyl-octane	C_9_H_20_	3	2216–33-3	-
4-oxohex-2-enal	C_6_H_8_O_2_	3	20697–55-6	-
5-ethyl-2(5H)-furanone ^Ψ^	C_6_H_8_O_2_	3	2407–43-4	**Sweet, spicy**
6-methyl-5-hepten-2-one ^Ψ^	C_8_H_14_O	2	110–93-0	**Fruity-like odor, pungent, green**
Acetic acid ^Ψ^	C_2_H_4_O_2_	3	64–19-7	**Fusty, vinegary, rancid**
Benzaldehyde ^Ψ^	C_7_H_6_O	2	100–52-7	**Almond, burnt sugar**
Benzeneethanol	C_8_H_10_O	2	60–12-8	**Honey-like**
Benzenemethanol	C_7_H_8_O	3	100–51-6	**Sweet, fruity**
Benzonitrile	C_7_H_5_N	3	100–47-0	-
Butyl-cyclohexane	C_10_H_20_	2	1678–93-9	-
Cyclobut-1-enylmethanol	C_5_H_8_O	3	89182–08-1	-
Decahydro-1,6-dimethyl-naphthalene	C_12_H_22_	3	1750–51-2	-
Decane ^Ψ^	C_10_H_22_	2	124–18-5	-
Ethyl acetate	C_4_H_8_O_2_	2	141–78-6	**Fruity, aromatic, sweet, winey**
Ethylbenzene ^Ψ^	C_8_H_10_	2	100–41-4	**Fruity**
Ethyl-hexanoate ^Ψ^	C_8_H_16_O_2_	2	123–66-0	-
Ethyl-pyrazine	C_6_H_8_N_2_	2	13925–00–3	-
Hexanal ^Ψ^	C_6_H_12_O	2	66–25-1	**Green, green apple, grassy, tallowy, leaf-like**
Hexyl-ethanoate ^Ψ^	C_8_H_16_O_2_	2	142–92-7	**Green, fruity, grass, sweet, apple**
Indane ^Ψ^	C_9_H_10_	2	496–11-7	-
Isobutyl-2-methyl-2-propenoate	C_8_H_14_O_2_	3	97–86-9	-
Methoxy-phenyl-oxime	C_8_H_9_NO_2_	3	-	-
Methylbenzoate	C_8_H_8_O_2_	3	93–58-3	**-**
Methyl-pyrazine	C_5_H_6_N_2_	2	109–08-0	-
n-hexylmethylamine	C_7_H_17_N	3	35161–70-7	-
Nonanal ^Ψ^	C_9_H_18_O	3	124–19-6	**Rancid with unpleasant and penetrating notes**
Nonane	C_9_H_20_	3	111–84-2	-
Octane	C_8_H_18_	2	111–65-9	**Solvent, unpleasant**
o-cymene	C_10_H_14_	2	527–84-4	**-**
Pentanal	C_5_H_10_O	3	110–62-3	**Almond-like, pungent, malt, woody, bitter, oily**
Pentyl-cyclohexane	C_11_H_22_	3	4292–92-6	-
Propyl-benzene ^Ψ^	C_9_H_12_	2	103–65-1	-
Propyl-cyclohexane ^Ψ^	C_9_H_18_	3	1678–92-8	-
Pyrazine	C_4_H_4_N_2_	2	290–37-9	-
Pyridine	C_5_H_5_N	2	110–86-1	-
Styrene	C_8_H_8_	2	100–42-5	**Balsamic, gasoline**
Sylvestrene ^Ψ^	C_10_H_16_	3	1461–27-4	-
Undecane	C_11_H_24_	2	1120–21-4	-
α-copaene ^Ψ^	C_15_H_24_	3	3856–25-5	**Woody**
β-myrcene	C_10_H_16_	2	123–35-3	-
β-pinene ^Ψ^	C_10_H_16_	3	18172–67-3	**Herbal, pine**

^Ψ^Most informative analytes across the samples for PLS-DA class discrimination

### Chemometric modelling

A peak table-based approach was adopted, enabling robust statistical and multivariate analysis with reduced computational demands. Data processing was performed using PLS-DA, a widely used supervised analysis method. The objective was to create a model capable of distinguishing between two classes: class 1 (defective oils: virgin and lampante virgin) and class 0 (non-defective oils: extra virgin).

**Fig. 2:**
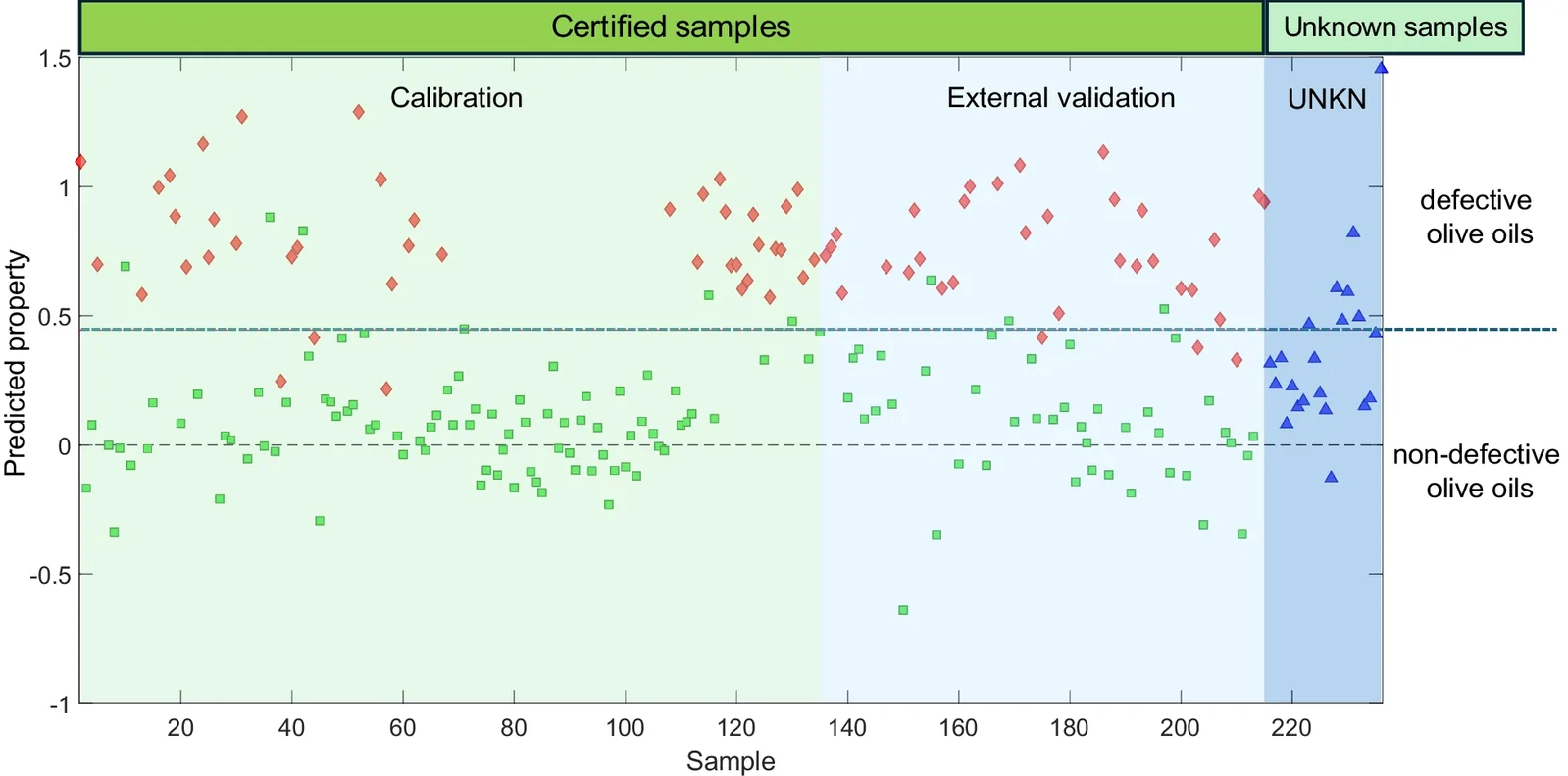
Classification of olive oil samples based on the predicted *y*-values from the PLS-DA model. Samples with predicted *y*-values lower than the threshold (dashed line at 0.45) are classified as belonging to the non-defective olive oil group. The threshold was determined with a 95% confidence interval using the PLS Toolbox. Samples with responses higher than the threshold are classified as defective olive oils. The symbols represent the actual class of each sample: yellow squares (■) for non-defective and red diamonds (♦) for defective oils. The blue triangles (▲) represent the undisclosed olive oil samples (UNKN), as described in the “Quality prediction of undisclosed olive oil samples” section. Readers are referred to the electronic version of this article for color interpretation

The number of LVs was selected based on the analysis of the calibration and cross-validation (Venetian blind) classification average errors. A PLS-DA model with 4 LVs was built. Sample classification depends on the predicted *y*-values for the training and external validation samples, as shown in Fig. 2. The model established a critical threshold of 0.45 (at a 95% confidence level) to distinguish between the classes. Samples with a predicted *y*-value below 0.45 were classified as non-defective, while those above the threshold were classified as defective. The performance of the final model was assessed using an external validation set, and the corresponding confusion matrix is presented in Table S2 (Electronic Supplementary Material). The model achieved adequate predictive accuracy of 93% and 91% in calibration and external validation, respectively, surpassing some reported performances in the literature (Table 2).

**Table 2:** A summary of studies that used different analytical approaches for the authentication and classification of virgin olive oils

Technique	Number of samples	Objective	Method and primary measure	Ref
HS-SPME-GC × GC–MS	215 (137 EVOO, 47 VOO, and 31 LVOO) + 21 undisclosed oils	Classification model (EVOO vs VOO / LVOO) using PLS-DA	Accuracy: 93% (calibration) / 91% (external validation). Sensitivity: 93% (calibration) / 89% (external validation)	This work
HS–SPME–GC–MS	176 (54 EVOO, 74 VOO, and 48 LVOO)	Classification model (EVOO vs VOO / LVOO) using PLS-DA	Sensitivity: 84%	[35]
HS-GC-IMS	94 (30 EVOO, 32 VOO, and 32 LVOO)	Classification (EVOO vs VOO / LVOO) using LDA, kNN, SVM	Accuracy: 83% (LDA) / 73% (kNN) / 88% (SVM)	[20]
HS–SPME–GC–MS	1796 (EVOO and non-EVOO (VOO and LVOO))	Classification model (EVOO vs VOO / LVOO) using *t-test-FwS-LDA*	Accuracy: 80%	[23]
Fluorescence and Raman spectrometry	112 (50 EVOO, 41 VOO, and 21 LVOO)	Classification model (EVOO vs VOO / LVOO) using PLS-DA	Sensitivity: 68% (fluorescence) / 70% (Raman)	[21]
FT-IR, NIR, and Raman Spectrometry	100 (35 EVOO, 49 VOO, and 16 LVOO)	Classification model (VOO and LVOO) using PLS-DA	Sensitivity: 92%	[7]
FT-IR, NIR, and HS-GC-IMS	112 (50 EVOO, 41 VOO, and 21 LVOO)	Classification model (EVOO vs VOO / LVOO) using PLS-DA	Sensitivity: 90% (MIR) / 93% (NIR) / 90% (HS-GC-IMS)	[36]
*Related applications*				
GC × GC–MS/FID	35 EVOO (2 cultivars)	Classification model (EVOO cultivars) using PLS-DA	Not available	[8]
HS–SPME–GC–MS	400 (246 EU and 154 non-EU virgin oil)	Classification model (geographical authentication of VOO) using PLS-DA	Sensitivity: 81% / Specificity: 95%	[22]
HS–SPME–GC–MS	82 EVOO (7 countries)	Classification model (geographical authentication of EVOO) using PLS-DA	Sensitivity: 73% (peak table-based) / 100% (pixel-based)	[26]
FGC E-nose	278 EVOO (Italian and non-Italian)	Classification model (geographical authentication of EVOO) using PLS-DA	Not available	[10]
GC-FID and GC–MS	225 VOOs (104 Cypriot and 121 Koroneiki cultivars)	Pattern recognition (PCA and HCA) of two VOO cultivars	Not applicable	[12]
NIR and fluorescence	72 EVOO (40 genuine and 32 adulterated)	One-class classification model using DD-SIMCA	Sensitivity: 95% (NIR) / 93% (Fluorescence) / 94% (data fusion of NIR + Fluorescence)	[13]
HS–SPME–GC–MS	320 VOO (9 cultivars)	Classification model using PLS-DA and LDA	Sensitivity (PLS-DA): 90% (calibration) and 81% (external validation) Sensitivity (LDA): 96% (calibration) and 93% (external validation)	[4]

*AHC* agglomerative hierarchical clustering, *EU* European Union, *FGC E*-nose flash gas chromatography electronic nose, *FT–IR* medium infrared spectrometry

As summarized in Table 2, several spectroscopic techniques have shown potential for virgin olive oil evaluation [7, 21, 36]. FT-IR produced promising classification results when compared to NIR and Raman spectrometry for binary classification between LVOO and non-LVOO samples (EVOO and VOO) [7]. Fluorescence spectrometry achieved an overall accuracy of 78% for ternary classification (EVOO, VOO, and LVOO), which was lower than that obtained with Raman spectrometry [21]. These accuracies were also below those achieved using gas chromatography–ion mobility spectrometry (GC-IMS) [20, 36]. Other techniques, such as GC–MS [35] and GC-IMS [20, 36], also provided satisfactory classification performance. Using support vector machines (SVM), researchers achieved an accuracy of 88%, slightly higher than that obtained with linear discriminant analysis (LDA, 83%) and k-nearest neighbors (KNN, 73%) for the classification of EVOO, VOO, and LVOO samples [20]. Overall, fluorescence and Raman spectrometry yielded the least accurate (or least sensitive) models, likely because their spectra have lower information content. In contrast, vibrational-based spectroscopic techniques such as FT-IR and NIR, together with GC-based methods, provided the best classification performance for differentiating EVOO, VOO, and LVOO samples.

In our GC×GC study (Fig. 2), some samples are located near the classification threshold, creating an “uncertainty region.” To address this, some researchers have proposed a hybrid strategy in which only borderline samples are sent for sensory analysis [35]. This approach reportedly reduced the tasters’ workload by 80% while maintaining the model’s high reliability (error rate below 10%).

Based on the satisfactory predictive performance shown in Table S2 (Electronic Supplementary Material), the PLS-DA model’s regression coefficients were used to identify the most discriminatory volatile compounds. This analysis, which resembles the analysis of PLS-DA VIP scores [37], assumes that variables with higher absolute coefficient values are more relevant for the model’s classification of samples as defective or non-defective.

**Fig. 3:**
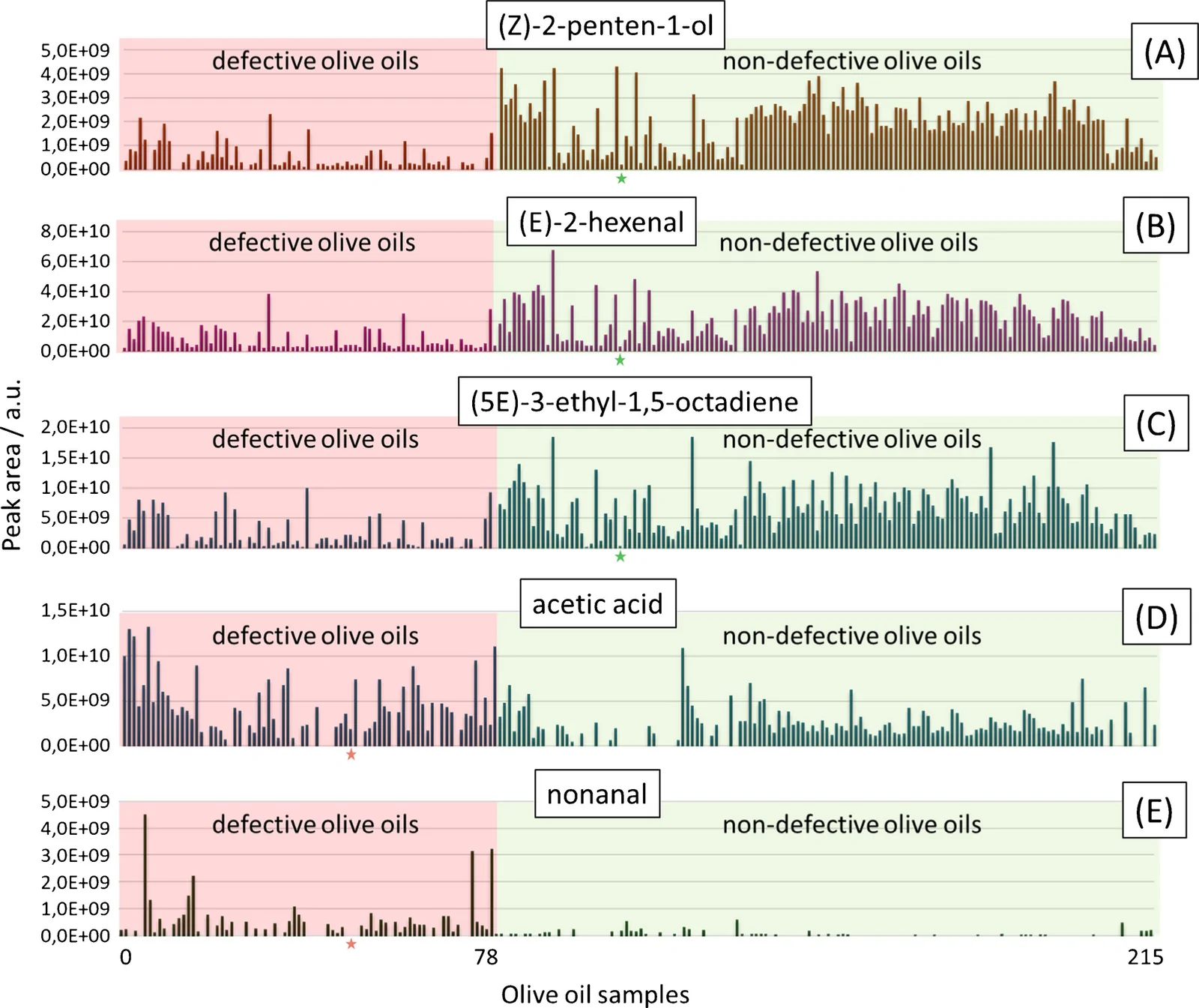
Distribution patterns for five of the most informative compounds for PLS-DA classification between non-defective and defective oils. Green stars highlight a misclassified non-defective sample, while red stars indicate a misclassified defective sample

Olive oil’s VOCs encrypt information about several key-quality attributes, including olive cultivar and their geographical origin, harvest stage, oxidation level, and sensory profile [10, 38, 39]. The analysis of the regression coefficients from the PLS-DA thus helps to identify the chemical markers driving the separation between non-defective and defective oils. The 39 most influential compounds identified by this method are highlighted in Table 1. Furthermore, Fig. 3 shows the distribution patterns of five of the most informative compounds, highlighting their differing abundances between the two classes.

Several compounds derived from the lipoxygenase (LOX) pathway were identified as key markers for the non-defective class. Among these, the C_5_ alcohol (Z)−2-penten-1-ol, associated with a positive green note, was detected at higher levels in non-defective samples (Fig. 3A), a trend previously reported for high-quality EVOOs [33]. Another one of the most informative analytes for the model was (E)−2-hexenal, a C_6_ aldehyde associated with positive fresh-green and fruity notes in olive oil [30]. As shown in Fig. 3B, the non-defective group exhibited a significantly greater abundance of this compound. Notably, this C_6_ aldehyde was also the most abundant analyte found in all the samples investigated in [33].

Furthermore, Fig. 3C illustrates the higher abundance of (5E)−3-ethyl-1,5-octadiene in the non-defective virgin olive oil group. Indeed, this hydrocarbon is known as a discriminative compound for earlier harvest stages during olive ripening [18, 28]. Other known early-ripening markers found in our work include (E,E)−2,4-hexadienal, (E)−2-pentenal, (E)−3-hexenal, (Z)−3-hexenal, and (Z)−2-hexenal [24, 28].

The only C_5_ ketone among the most informative analytes in the PLS-DA model was 1-penten-3-one. This odor-active volatile compound is formed through the LOX route [4, 18], and, similar to other C_5_ and C_6_ alcohols and ketones, it may carry information that can be used for authentic EVOO assessment, depending on the sample set [16]. Another LOX-derived compound, 1-penten-3-ol, is linked to desirable green and pungent sensory notes [1]. Its concentration is known to decrease as olives ripen [28, 40], a trend consistent with our results, where 1-penten-3-ol was more abundant in the non-defective oils, which are typically produced from olives at earlier maturation stages.

Conversely, the defective oil group showed elevated levels of compounds linked to sensory defects and advanced maturation, including acetic acid and nonanal. Acetic acid, shown in Fig. 3D, is a known contributor to vinegary notes [16]. Nonanal (Fig. 3E) not only imparts a harsh, rancid off-flavor, but its concentration is also known to increase throughout olive oil storage [25, 33]. The formation of this aldehyde derives from the metabolism of fatty acids, specifically the homolytic cleavage of 13-hydroperoxides of linoleic acid and 9- and 10-hydroperoxides of oleic acid [1]. Literature also mentions compounds known to be discriminative for the late harvest stages of olives, including 1-octanol and 6-methyl-5-hepten-2-one [18, 28]. Interestingly, an unexpected behavior was observed for the latter, which was generally more abundant in the non-defective virgin olive oil group. The 1-octanol is an alcohol typically found in greater abundance in olive oils produced from overripe olives, which are already undergoing oxidation [18, 39, 41]. This C_8_ alcohol imparts a negative mushroom-like off-flavor, degrading the overall sensory profile [24, 29]. Accordingly, it was found to be significantly higher in the defective oil group of our sample set.

As potential markers for the genetic and geographical origin of EVOO and VOO [11, 42–45], some terpenes tentatively identified in our work and previously reported on olive oil include β-pinene (herbal, pine), α-copaene (woody), sylvestrene, (E)-β-ocimene, and (E)−4,8-dimethylnona-1,3,7-triene [29, 46]. Although the last one did not present a discriminative distribution pattern between our sample groups, it was previously identified with an abundance > 30% in Brazilian olive oils when compared to Italian ones [29]. Interestingly, even though our sample set comprised virgin olive oils from several regions of Brazil, (E)-β-ocimene was elevated in the defective virgin olive oil group, suggesting that this terpene warrants further attention as a promising indicator for differentiating quality grades in Brazilian virgin olive oils.

In addition to (Z)−2-penten-1-ol, other discriminant alcohols in the PLS-DA model include 1-hexanol, 1-penten-3-ol, and (E)−2-hexen-1-ol. The two C_6_ alcohols, 1-hexanol and (E)−2-hexen-1-ol, are likely derived from the LOX cascade via the partial reduction of their respective aldehyde precursors. Both were previously identified as relevant compounds for Brazilian olive oils and were among the 12 most indicative predictors for discriminating them from Italian ones [29, 47].

Finally, it is worth investigating the characteristics of the samples misclassified by the PLS-DA model. The abundances of the five most informative compounds for two misclassified virgin olive oils (one defective and one non-defective) are highlighted by colored star-shaped markers in Fig. 3.

As shown in Fig. 3A–C, the abundance (green stars) of three key quality markers in the misclassified non-defective sample deviated from expected levels. Instead of exhibiting high peak areas for (Z)−2-penten-1-ol, (E)−2-hexenal, and 5(E)−3-ethyl-1,5-octadiene, this sample showed very low peak areas of these desired compounds. This behavior likely contributed to the PLS-DA model misclassifying this non-defective sample as defective.

A similar deviation is observed in Fig. 3D, E for the misclassified defective sample. The red stars highlight unexpected low abundances or the absence of acetic acid and nonanal, compounds typically associated with defects. This likely led the model to assign the defective sample to the non-defective group incorrectly.

However, it must be emphasized that attempting to rationalize a sample’s classification solely by inspecting the concentration of individual compounds has inherent limitations. The PLS-DA model is multivariate, and its predictions depend on linear combinations of the original variables (i.e., peak areas), accounting for subtle interplay and synergistic or antagonistic effects between variables. Consequently, the peak area of a single compound is rarely the sole determinant of a classification. Therefore, the analysis of individual compounds should be considered purely illustrative, offering insight into the complex patterns driving the model rather than serving as a univariate justification for its predictions. This same principle applies to the discussion in the “Quality prediction of undisclosed olive oil samples” section.

### Quality prediction of undisclosed olive oil samples

As previously mentioned, the initial dataset comprised a subgroup of virgin olive oils with previously assessed quality and 21 undisclosed samples whose quality was unknown (henceforth, UNKN samples). In this work, the trained PLS-DA model was used to predict the quality of these 21 UNKN samples. Based on this procedure, seven UNKN samples were expected to be defective, and the remaining 14 were classified as non-defective (Fig. 2).

**Fig. 4:**
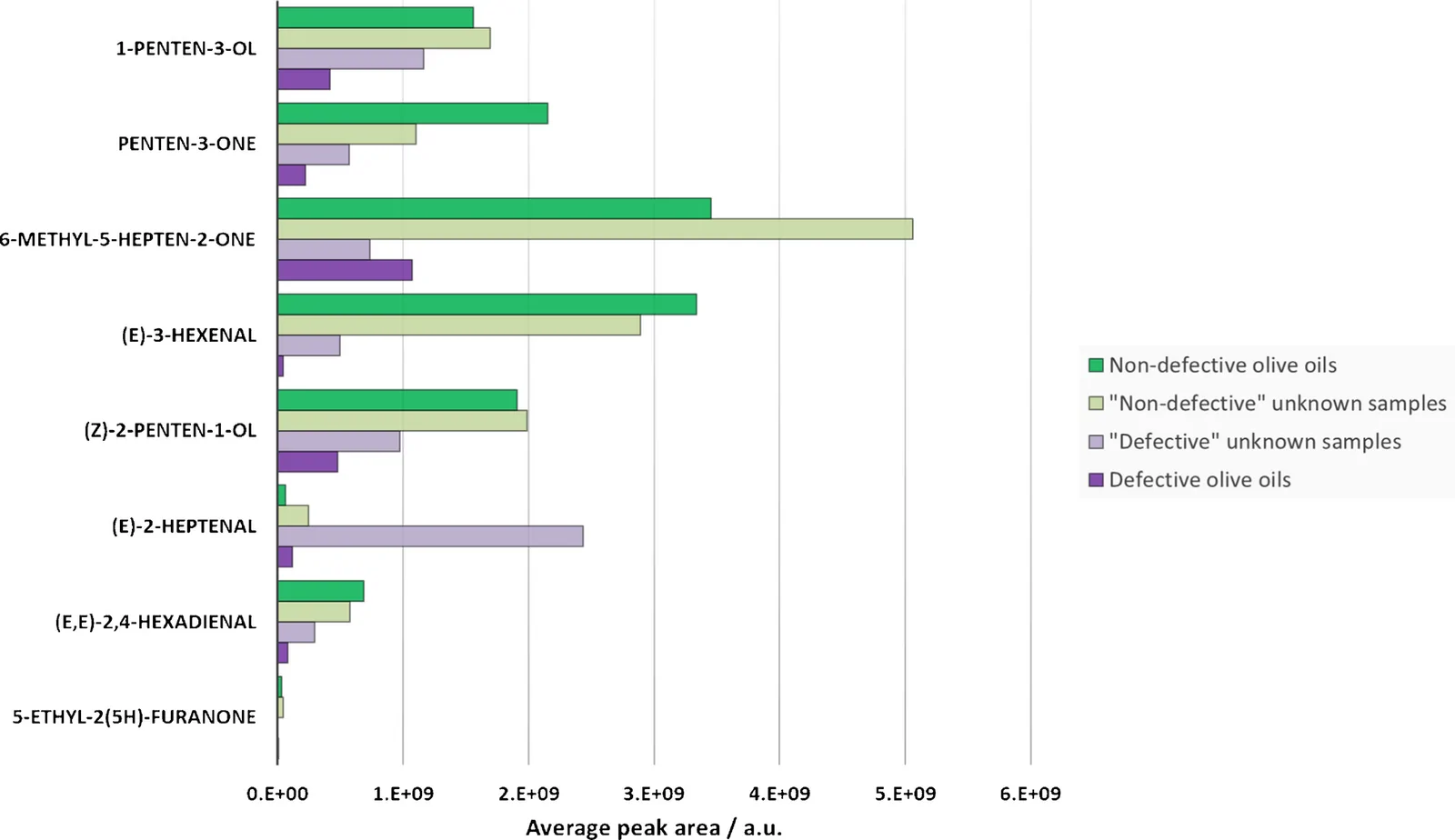
Average peak areas of volatile compounds across the original “defective” and “non-defective” oils and the 21 undisclosed samples predicted into these same classes

Figure 4 illustrates the average peak area of volatile compounds across four groups: the original “defective” and “non-defective” oils, and the samples (UNKN) predicted into these same classes. The results show strong alignment between the chemical profiles of the original samples and those of the newly classified UNKN.

Several compounds previously identified as quality markers were analyzed. Notably, 1-penten-3-ol, (Z)−2-penten-1-ol, penten-3-one, 6-methyl-5-hepten-2-one, and (E)−3-hexenal show markedly higher abundances in both the known non-defective oils (dark green) and the non-defective UNKN (light green) compared to their defective counterparts. This consistency suggests that the model correctly identified this chemical signature, which is often associated with desirable “green” and “pungent” sensory notes, and used it to classify the new samples.

Conversely, (E)−2-heptenal was more abundant in the original defective virgin olive oil group than in the non-defective one. This same pattern was observed across the UNKN groups, confirming the model’s ability to associate this compound, known to cause rancid off-flavors, with the defective category. Overall, the consistent chemical patterns between the known oils and the predicted UNKN provide promising evidence for the model’s ability to accurately classify new, unseen virgin olive oils produced in the year 2024.

## Conclusions

The present study developed a robust and non-subjective analytical method that integrates HS-SPME-GC×GC–MS with chemometric analysis for the quality assessment of Brazilian olive oils. The PLS-DA model achieved 93% (calibration)/91% (external validation) accuracy in classifying samples as “defective” (VOO and LVOO) or “non-defective” (EVOO) based on their volatile chemical profiles. Examination of regression coefficients revealed key volatile markers associated with oil quality. High-quality, non-defective oils were characterized by an abundance of lipoxygenase (LOX) pathway compounds, such as (E)−2-hexenal and (Z)−2-penten-1-ol, responsible for desirable “green” and “fruity” notes. In contrast, defective oils exhibited higher levels of nonanal and acetic acid, compounds linked to oxidation and microbial activity that led to rancid sensory attributes. The model was applied to 21 undisclosed samples, which showed consistent alignment between chemical profiles and model-based predictions. Overall, this work demonstrates that the VOC fraction provides an objective and reliable basis for virgin olive oil classification, serving as a valuable complement to traditional sensory evaluation. This instrumental approach offers a robust and interpretable alternative for forensic analysis of virgin olive oils in LFDAs, enabling models that translate directly into sensory-relevant insights.

## Supplementary Information

Below is the link to the electronic supplementary material.

Supplementary file1 (DOCX 23.1 KB)

## Data Availability

The datasets used in this study are available from the corresponding author upon reasonable request.
