# Supplementary material for: Quality assessment of Brazilian olive oils by GC×GC–MS and chemometrics
Source: Anal Bioanal Chem. 2026 Jan 27;418(10):2999–3011. doi: 10.1007/s00216-026-06321-8 (PMC13144221; doi:10.1007/s00216-026-06321-8)
Supplement: Supplementary file 1 — Supplementary file1 (DOCX 23.1 KB) [file 216_2026_6321_MOESM1_ESM.docx]

***SUPPLEMENTARY MATERIAL FOR***

**Quality assessment of Brazilian olive oils**

**by GC×GC-MS and chemometrics**

Andre Cunha Paiva^1,2^, Glaucimar Alex Passos de Resende^1,2^, Luidy Darllan Barbosa^1^, Daniel Lucas Dantas Freitas^3^, Guilherme Post Sabin^3^, Leandro Wang Hantao^1,2,4*^

^1^Instituto de Química (IQ), Universidade Estadual de Campinas, Campinas, SP, Brasil.

^2^Instituto Nacional de Ciência e Tecnologia (INCTBio), Campinas, SP, Brasil.

^3^OpenScience, Campinas, SP, Brasil,

^4^Núcleo Interdisciplinar de Planejamento Energético (NIPE), Campinas, SP, Brasil

**Supplementary Material**

**Table S1.** Distribution of the virgin olive oil samples into extra virgin, virgin, and lampante virgin categories.

|  | Number of samples per category |
| --- | --- |
| Extra virgin olive oil | 137 |
| Virgin olive oil | 47 |
| Lampante virgin olive oil | 31 |

**Table S2.** Confusion matrix for the prediction results (external validation) of the PLS-DA model constructed with 4 LVs. Note: confusion matrix values were based on the "most probable" prediction classification.

|  | **Real class** | |
| --- | --- | --- |
|  | defective olive oils | non-defective olive oils |
| **Predicted as defective olive oils** | 25 | 3 |
| **Predicted as non-defective olive oils** | 3 | 37 |
| **Unassigned** | 0 | 0 |
